# Microglial inflammation in the parkinsonian substantia nigra: relationship to alpha-synuclein deposition

**DOI:** 10.1186/1742-2094-2-14

**Published:** 2005-06-03

**Authors:** Emilie Croisier, Linda B Moran, David T Dexter, Ronald KB Pearce, Manuel B Graeber

**Affiliations:** 1 Department of Neuropathology, Division of Neuroscience and Mental Health, Imperial College London, and Hammersmith Hospitals Trust, London, UK; 2 Department of Cellular and Molecular Neuroscience, Division of Neuroscience and Mental Health, Imperial College London, London, UK

## Abstract

**Background:**

The role of both microglial activation and alpha-synuclein deposition in Parkinson's disease remain unclear. We have tested the hypothesis that if microglia play a primary role in Parkinson's disease pathogenesis, the microglial "activated" phenotype should be associated with histopathological and/or clinical features of the disease.

**Methods:**

We have examined microglial MHC class II expression, a widely used marker of microglial activation, the occurrence of CD68-positive phagocytes and alpha-synuclein immunoreactivity in post-mortem human substantia nigra affected by idiopathic Parkinson's disease (PD). Using semi-quantitative severity ratings, we have examined the relationship between microglial activation, alpha-synuclein deposition, classical neuropathological criteria for PD, subtype of the disease and clinical course.

**Results:**

While we did not observe an association between microglial MHC class II expression and clinical parameters, we did find a correlation between disease duration and the macrophage marker CD68 which is expressed by phagocytic microglia. In addition, we observed a significant correlation between the degree of MHC class II expression and alpha-synuclein deposition in the substantia nigra in PD.

**Conclusion:**

While microglia appeared to respond to alpha-synuclein deposition, MHC class II antigen expression by microglia in the substantia nigra cannot be used as an indicator of clinical PD severity or disease progression. In addition, a contributory or even causative role for microglia in the neuronal loss associated with PD as suggested by some authors seems unlikely. Our data further suggest that an assessment of microglial activation in the aged brain on the basis of immunohistochemistry for MHC class II antigens alone should be done with caution.

## Introduction

Parkinson's Disease (PD) is a common neurodegenerative disorder with the cardinal clinical features of tremor, rigidity, bradykinesia and loss of postural reflexes. Neuropathologically, the disease is characterized by a marked loss of dopaminergic neurons in the substantia nigra pars compacta (SN) and the presence of alpha-synuclein (aSN)-positive Lewy bodies (LBs) in neurons of this and other brain areas also affected by nerve cell death. An international consensus definition of Lewy body diseases on the basis of molecular as well as morphological criteria, which takes into account aSN status of the brain, has been published recently [1].

The discovery of aSN mutations and gene amplification in some familial forms of PD [2-6]] and the identification of this protein as a major component of Lewy bodies (LBs) in common sporadic PD [7], has spurred interest in the role of aSN in the pathophysiology of PD and other synucleinopathies. However, no direct causal relationship has yet been established between aSN aggregation and the selective neuronal cell death characteristic of PD. LBs are also found incidentally in aged brain in the absence of other pathological features and without a clinical history of parkinsonism or dementia [8]. Attempts have been made to link the clinical progression of PD to the presence of aSN inclusions and an anatomical staging model has been proposed [9], but the latter has been questioned by subsequent studies in which clinical data were also taken into account [10].

Apart from well established morphological criteria, activated microglia can be defined in tissue sections on the basis of the expression of several immune function-related proteins, notably complement receptors and MHC class II antigens (MHCII). Phagocytic activity and cytotoxic properties are usually considered end stages of microglial activation, at which point they are phenotypically indistinguishable from blood-borne macrophages. Activated microglia are associated with a large range of neurological insults from trauma and infection to autoimmune conditions, and their presence represents a common finding also in neurodegenerative disorders [11]. However there is little knowledge about the molecular processes that mediate microglial activation and exactly which biological consequences may result from their enhanced state of "immune alertness" within affected CNS tissue. A transcriptome signature of interferon-gamma activated microglia has been provided recently [12]. Microglial phenotypic changes have also been observed in normal aged individuals [13]. Thus, "microglial senescence" confounds the problem of a definition of microglial activation in disease states, and in neurodegenerative diseases in particular which are often age-related, as no specific causative stimulus has been identified in the process.

While microglia clearly show changes in their phenotypic profile in neurodegeneration, it is by no means clear whether they are actively involved in the progression of PD. Microglia-derived macrophages can be found in the PD SN, and neuromelanin pigment taken up from degenerated dopaminergic nerve cells is characteristically observed in SN phagocytes. In animal models of nigrostriatal degeneration using 6-hydroxydopamine and 1-methyl-4-phenyl-1,2,3,6-tetrahydropyridine (MPTP), inhibition or attenuation of the microglial immune response increases neuronal survival. However, those results have so far not been replicated in clinico-pathological studies, and the simple chemical lesions currently employed in animal studies by all likelihood do not fully reflect the chronic neurodegenerative disease process in humans [14].

In the present study, we independently evaluate the severity of alpha-synuclein deposition and microglial activation identified by immunohistochemical staining in the SN in a large cohort of clinically and pathologically confirmed PD cases. We have studied the microglial response in PD on two levels, by observing MHCII -immunoreactive cells (putatively activated microglia but possibly only senescent cells) and CD68-immunopositive macrophages (corresponding to either phagocytic microglia or cells derived from invading blood-borne macrophages).

## Materials and methods

### Parkinson's disease cases

37 PD nigrae were evaluated immunohistochemically. 20 cases were provided by the UK Parkinson's Disease Society Tissue Bank at Imperial College London (PDSTB). Additional tissue sections from 17 other cases came from a previous study originally performed at the Institute of Neuropathology, University of Munich, Germany. These Parkinson's cases had been previously diagnosed, neuropathologically screened for confounding pathology, and examined in a study of apoptosis and microglial activation [15]. Archival sections were immunolabelled for alpha-synuclein (see below) and used as a control group to ensure that variation within our PDSTB cohort was within an established range.

### Clinical and neuropathological assessment of cases

For the PDSTB cohort, clinical reports were evaluated in detail by an experienced neurologist with a special interest in Parkinson's disease (RKBP). Neuropathological assessment was based on slides provided by the PDSTB for alpha-synuclein, tau and beta-amyloid immunohistochemistry of superior frontal gyrus, the hippocampal region and midbrain as minimum data sets, and screening of the cases for confounding pathology was based on hematoxylin and eosin examination of a standard series of 18 tissue blocks following a standardised dissection procedure [16]. Nine cases showed varying degrees of concurrent Alzheimer's disease (AD)-type pathology (tau-immunopositive tangles and/or beta-amyloid-immunopositive plaques) of isocortical and/or entorhinal type ranging from grades 1–3 . Three cases were excluded based on a final neuropathological diagnosis of AD, progressive supranuclear palsy (PSP), and young-onset familial PD, respectively, leaving a cohort of 17 cases in each the Munich and PDSTB groups (Table 1).

**Table 1:** PDSTB cases examined.

**CASE**	**SEX**	**DIAGNOSIS**	**AAO**	**AAD**	**DD**	**MHCII AVE**	**aSN AVE**	**CD68 AVE**
1	m	PD, H-T	63	72	9	2	1	1.5
2	f	PDD H-T	67	81	14	2	1	1
3	m	PD, A-R	57	71	14	2.75	3	2.25
4	f	PD, H-T	67	85	18	2.5	2.25	0.5
5	m	PD, H-T	57	75	18	2	1.5	2
6	m	PD, A-R	78	83	5	1.75	1	2.75
7	f	PD, H-T	55	73	18	1.75	2	2.25
8	m	PD, H-T	49	77	28	2.5	2.5	0.5
9	f	PD, H-T	65	75	10	2	2	2.75
10	m	PD, A-R	75	82	7	1	1.75	2
11	m	PDD H-T	72	81	9	2	2	1.25
12	m	PD, A-R	69	75	6	2	2.5	1.75
13	m	PD, A-R	65	83	18	2.5	1.5	1.5
14	m	PD, H-T	70	77	7	2	1	1.5
15	m	PD, A-R	86	89	3	2.75	2.5	2
16	f	PD, H-T	72	83	11	2	1.5	1.25
17	m	PD, H-T	66	76	10	2.5	2.5	3

Abbreviations: PD, Parkinson's disease; PDD, Parkinson's disease with dementia; H-T, hemi-tremulous; A-R, akinetic-rigid; AAO, age at disease onset; AAD, age at death; DD, disease duration; AVE, semi-quantitative severity rating, averaged across two observers.

### Immunohistochemical evaluation of protein levels

Immunohistochemical reactions were performed using the avidin-biotin complex (ABC)/peroxidase method with mouse monoclonal antibodies anti-human HLA-DP, DQ, DR (clone CR3/43, Dako, dilution 1/100) and anti-alpha-synuclein (Becton-Dickinson, dilution 1/300). For the PDSTB group, additional immunohistochemistry was carried out with anti-CD68 (clone PGM1, Dako, dilution 1/200). Sections were dewaxed in xylene, rehydrated, and endogenous peroxidase activity was blocked by 30 min exposure to 1% hydrogen peroxide in methanol. Antigen unmasking consisted of boiling in 0.01 M EDTA (20 min. at 350 W in microwave) and 100% formic acid treatment (3 min.) prior to incubation with anti-HLA-DP, DQ, DR and anti-alpha-synuclein, respectively. No antigen unmasking was used with anti-CD68. Slides were then incubated in primary antibody diluted in phosphate-buffered saline (PBS) overnight at 4°C. The following day, after washing in PBS, they were incubated in horse-anti-mouse secondary antibody (Vector, dilution 1/200) and finally in ABC complex (Vector, dilution 1/200) each for 1 hour at room temperature. Immunoreactivity was visualised with 3,3'-diaminobenzidine (Vector kit).

After immunohistochemical staining, sections were given semi-quantitative severity ratings for aSN, MHCII, and CD68 immunoreactivity by two investigators (EC and MBG) blinded to case number. The SN was defined as the area extending laterally from the exit of the third nerve, superior to the cerebral peduncle and inferior to the medial lemniscus, ideally at the height of the red nucleus with the presence of melanised neurons or their remnants indicating the main region of interest. The severity ratings were determined across the entire region of SN, based on the density of immunopositive structures, with 0 (none), 1 (mild), 2 (moderate) and 3 (high). For aSN, both intra- and extra-cellular inclusions were considered provided they fell within the immediate area of the substantia nigra. This was particularly relevant in areas of severe neuronal loss, often encountered more laterally, where significant alpha-synuclein pathology could still be observed. The morphological variation in aSN deposition was not assessed, simply the frequency of events. All clearly identifiable aSN-immunoreactive structures, including LBs, neurites, fibrils, and smaller, punctate formations, were considered. For microglial response, severity was judged primarily by immunoreactivity, however morphology was taken into account in that perivascular immunoreactivity was excluded. The thickening of microglial processes increased the apparent density of microglial staining, such that cases undergoing a more intense microglial response were clearly differentiated on the basis of immunoreactivity alone. Morphological features of activated microglia were always noted, however there were *no *cases for which the severity rating would have changed substantially on the basis of morphological features, ie. cases with low MHCII immunoreactivity but most microglia adopting an amoeboid morphology or cases with high MHCII immunoreactivity but most microglia appearing ramified.

Regression analysis revealed the two sets of ratings from independent observers were highly correlated (p < 0.0001). Ratings were then averaged to generate a severity score for each immunohistochemical stain.

## Results

**Figure 1:**
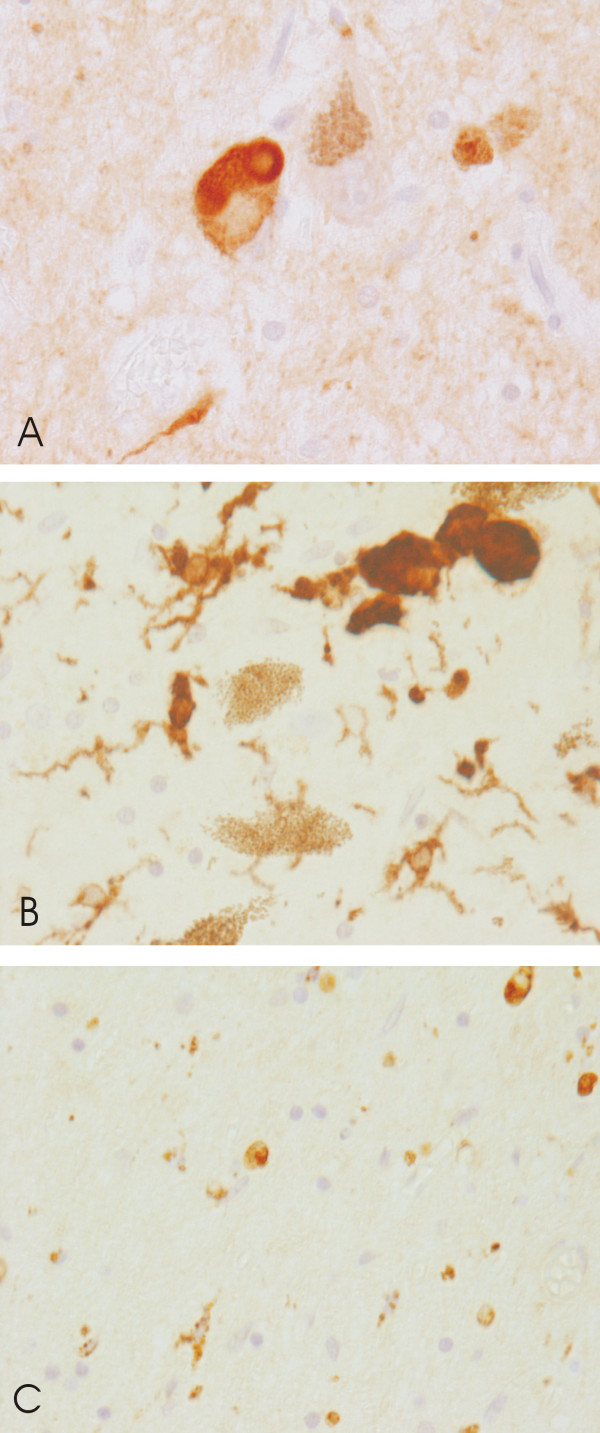
Immunohistochemical staining of (A) aSN deposition in and around melanised dopaminergic neurons of the SN, (B) microglial expression of MHCII, and (C) macrophage expression of CD68. All images taken at 40X primary magnification.

**Figure 2:**
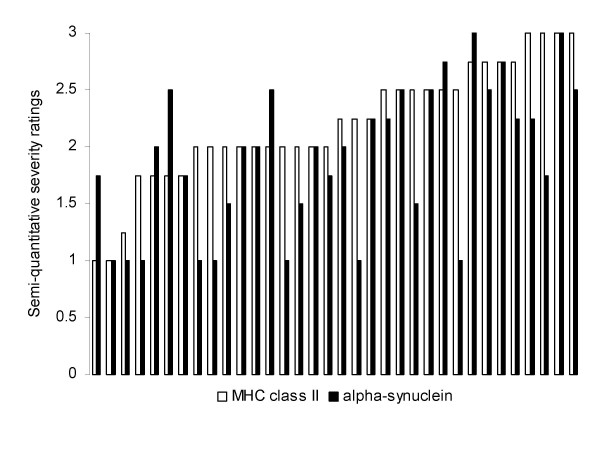
Case-by-case semi-quantitative severity ratings for MHC class II and alpha-synuclein immunopositivity.

All of the cases examined showed the severe dopaminergic neuronal loss and extra-cellular (free-lying) neuromelanin typical of advanced PD. All were also positive for alpha-synuclein, MHCII, and CD68 (Figure 1). Semi-quantitative ratings for the PDSTB cohort are shown in Table 1. MHCII immunoreactivity was confined to microglial or macrophage-like cells. Most of the CD68-positive cells were of a brain macrophage phenotype, i.e. cells with an enlarged immunoreactive cytoplasm containing lysosomal structures and/or neuromelanin degradation products, shortened and less ramified, stout (compared with typical microglia) cell processes but rarely of the appearance of the full-blown macrophages commonly found in brain infarcts or multiple sclerosis lesions. aSN inclusions were observed in neurons, white matter, and occasionally glial cells and their fine processes. MHCII immunolabelling and the presence of macrophages showed significant variation between cases. Semi-quantitative ratings revealed that despite this inconsistency across the group, aSN deposition and MHCII immunoreactivity were found to correlate within individual cases (p < 0.001) (Figure 2). A General Linear Model test (SPSS) revealed no difference between PDSTB and University of Munich cases (p = 0.01), and the relationship remained statistically significant when we considered only the PDSTB group. We did not find a similar relationship between aSN and CD68 immunoreactivity. Regression analysis revealed no significant statistical link between the two stains.

In our cohort of PDSTB cases (n = 17), for which clinical information was available, we then assessed how immunoreactivity may relate to clinical history. Cases were assessed based on clinical subtype (tremor or akinetic-rigid), gender, disease duration (DD), and ages of onset (AAO) and death (AAD). In addition, absence or presence of AD-related pathology was determined following ICDNS criteria . Cases were evaluated as individual data points and in groups. No correlations were found with aSN or MHCII as histological reference points, and no clinical classification seemed to reflect the relationship between aSN deposition and MHCII immunoreactivity in individual cases. A significant difference between CD68 immunoreactivity in PD cases with a DD of 10 years or less (n = 9, mean 7.3 ± 2.4 years) compared to those with a DD greater than 10 years (n = 8, mean 17 ± 5.0 years) was detected when using Student's t-test, with CD68 immunoreactivity significantly higher in cases with a shorter DD (p < 0.05). DD was highly inversely correlated with AAO (p < 0.0005). AAO was also significantly different in the two DD groups according to Student's t-test (p < 0.005) while AAD remained consistent across cases in both the shorter- (n = 9, mean 78.9 ± 5.3) and longer DD groups (n = 8, mean 78.5 ± 5.2). MHCII and aSN immunoreactivity did not show any significant variation across DD, or any other clinically defined PD groups.

## Discussion

Our finding of an overall correlation between aSN deposition and MHCII-expressing microglia in the substantia nigra is in line with the finding that both phenotypic changes are associated with neurodegeneration in PD, but it remains unclear whether there is any pathogenetic link. It is perhaps more noteworthy than the correlation between alpha-synuclein deposition and microglial activation that we failed to find any correlation between these parameters and clinical indicators of disease progression.

Studies of multiple system atrophy (MSA), PSP and corticobasal degeneration (CBD) have previously detected a stochastic link between the presence of activated microglia and protein deposition in the neuroanatomic systems specifically affected by the disease and hypothesize that microglial activation may in part be induced by the accumulation of pathological protein in tissue [17,18]. aSN could be one of the pathological substrates that initiate microglial activation. However, there is no evidence that LBs can directly provoke this response [19,20]. LBs may contain complement proteins and chromogranin A [21], which can induce microglial activation *in vitro *[22], but in post-mortem tissue microglia have not been observed to interact preferentially with these particular LBs [19].

Much has been speculated about the potentially deleterious effects of activated microglia on neuronal survival in PD. Specifically, emphasis has been placed on the production of pro-inflammatory cytokines and reactive oxygen species potentially increasing oxidative stress on surrounding neurons. In various animal and cell culture models, the inhibition of microglial activation has been demonstrated to be neuroprotective in some circumstances [23,24]. However, there is no evidence that microglia initiate neurodegeneration, and their response does not always correlate to active cell death occurring in their microenvironment. The presence of MHCII-immunoreactive microglia in the SN of monkeys one year following chronic administration of MPTP has been interpreted as evidence that the neurodegenerative process was still active and associated with the glial response [25]. However, a comparable experiment demonstrated that although microglia do indeed remain present in the SN, they are absent from the striatum where active neurodegeneration could still be detected [26].

In contrast to the focal activation observed in animal models and acute CNS insults, microglial activation in PD is widespread and not limited to areas of marked cell death [27]. This phenomenon, and the persistence of activation in the SN long after most dopaminergic neurons have been lost, may in part be attributable to the differences in microglial responsiveness between young and aged individuals. The number of MHCII-expressing microglia in the human CNS increases steadily with age independent of disease or trauma [13,28]. In addition, microglia and astrocytes in culture harvested from older rats are more inclined to proliferation and MHCII expression and are less sensitive to transforming growth factor-beta than glia from younger donors [29]. This is in line with the findings of a study on MPTP-treated mice showing that this toxin acts in an age-dependent manner, with protracted microglial activation in older animals [30].

Our failure to find any correlation between MHCII in the parkinsonian nigra and DD, AAO, AAD, gender or predominant motor symptoms raises questions about the significance of microglial MHCII expression as a marker of their involvement in PD and in other chronic CNS diseases especially in the aged brain. Are microglia designated 'active' by the presence of certain proteins necessarily functionally active? Or does microglial MHCII expression serve as a "firewall" against T-cell invasion of already compromised CNS tissue since co-stimulators such as B7 may not be expressed at a sufficient level [31]? The use of MHCII immunoreactivity as a marker of microglial "activation" should be re-evaluated in the light of studies suggesting very long-lasting microglial involvement in chronic and late onset neurological conditions. Microglial "activation" under such conditions may have the quality of a "microglial scar" which would have different functional relevance, and this should be taken into account in the interpretation of neuroimaging studies of activated microglia [32].

Furthermore, it may also be that the presence of activated microglia is a reflection of agonal state rather than indicative of a chronic disease state, and that our microglia results are in part attributable to factors such as hypoxia or infection prior to patient death. This could account for our failure to identify clinical correlates to MHCII expression; however the strong correlation of this expression with the presence of aSN deposition remains unexplained. Another possibility is that the inflammatory response in PD peaks early in the course of the disease, at which time a pathogenetic link between MHCII and clinical progression could be detected. However, by the time of post-mortem evaluation most relevant microglial activity may have stopped. Yet, high levels of CD68 in some cases indicate that microglial phagocytosis can still occur at the time of death. As the presence of tissue macrophages may be considered a sign of ongoing tissue destruction, and macrophages are known to be crucial players in the cytotoxic phase of an inflammatory response, it is of particular interest that they were found to be more prevalent in PD cases with shorter DD. This suggests that microglial phagocytosis may not persist when it is no longer functionally relevant.

Increased extraneuronal neuromelanin and decreased aSN pathology in the SN have been associated with the progression of PD as defined by a staging model based on pathological, rather than clinical criteria [9]. It is possible that as PD progresses from one pathological tier to another, increasing extracellular neuromelanin deposition causes more microglia to adopt a phagocytic phenotype. Neuromelanin has been demonstrated to induce microglial activation *in vivo *[33], and, since one of the primary functions of activated microglia is the removal of debris produced by necrotic cells and neuromelanin is a readily identifiable component of this debris, the presence of this compound in the SN may very well have a relationship with the presence of phagocytic microglia in the region. However, this pathological staging is not reflective of our disease cohort. All of the cases evaluated for this study were in both clinically and pathologically advanced stages of PD, and would have fallen within the later, more severe proposed tiers. Because the model does not address clinical information regarding disease progression, the distribution of DD across our cases would not affect their pathological staging. Cases with shorter DD may have progressed more rapidly, or they may have remained presymptomatic for a longer period. Our failure to find any relationship between aSN deposition and DD does not contradict observations that LBs decrease as the disease progresses, it merely supports the assertion that our cohort was entirely situated in the most severe stages of the disease. Extraneuronal neuromelanin would be expected, and was observed, throughout our cohort. Our observation that an increase in CD68 immunoreactive, putatively phagocytic microglia is correlated with a shorter DD provides a clinical refinement beyond the scope of a model based exclusively upon pathological observations. Whether microglial phenotype is a direct result of increased neuromelanin deposition does not affect the significance of our finding that it is related to DD.

Regardless of how it is induced, microglial phagocytosis of neural debris is not a rapid process [34,35] and this peculiarity of the brain's intrinsic phagocytes may provide the most straightforward explanation for the persistence of some CD68-positive cells in the SN even after a long disease course. Alternatively, there may be a difference between early- and late-onset cases of PD with respect to their formal pathogenesis, with earlier onset cases having a lower level of phagocytosis throughout the degenerative process. Parkinsonian-type, age-related neurodegeneration, diagnosed as idiopathic PD, observed in late-onset cases may share clinical symptoms with true idiopathic PD in spite of causative, prognostic, or pathogenic differences.

In conclusion, this study demonstrates that throughout the SN, PD cases with relatively high levels of aSN deposition can be expected to contain higher numbers of MHCII positive microglia but there is no correlation with specific clinical subtypes or symptoms. We also report that, unlike CD68-expressing macrophages, neither aSN deposition nor microglial MHCII is indicative of the duration of the disease course. Both aSN deposition and microglial MHCII expression are likely to hold some as yet unknown functional significance in the progression of PD, and their careful localization and characterization throughout the brain will help to shed light on their specific role in the disease process. However, attempts to link alpha-synuclein deposition or microglial activation with the clinical course of PD should be made with caution. Our finding that CD68 immunoreactivity correlates negatively with disease duration suggests that there may be a pathogenic difference between earlier and later-onset PD. Follow-up studies addressing genomic, transcriptomic, and proteomic differences, possible drug interactions and specific clinical correlates are needed.

## List of abbreviations

AAD, age at death; AAO, age at onset; AD, Alzheimer's disease; A-R, akinetic-rigid; aSN, alpha-synuclein; DD, disease duration; H-T, hemi-tremulous; LB, Lewy bodies; MHCII, major histocompatibility complex class II; MPTP, 1-methyl-4-phenyl-1,2,3,6-tetrahydropyridine; MSA, multiple systems atrophy; PD, Parkinson's disease; PDD, Parkinson's disease with dementia; PDSTB. Parkinson's Disease Society Tissue Bank; PSP, progressive supranuclear palsy; SN, substantia nigra.

## Competing interests

The author(s) declare that they have no competing interests

## Authors' contributions

MG and EC designed this study. EC did most of the lab work and wrote major parts of the paper. The data analysis was done jointly by EC, MG, RP and LM where indicated. DD played a crucial role in the provision of the necessary case material and contributed to the writing.
